# Long-term results after transplantation of pediatric liver grafts from donation after circulatory death donors

**DOI:** 10.1371/journal.pone.0175097

**Published:** 2017-04-20

**Authors:** Rianne van Rijn, Pieter E. R. Hoogland, Frank Lehner, Ernest L. W. van Heurn, Robert J. Porte

**Affiliations:** 1 Section Hepatobiliary Surgery and Liver Transplantation, Department of Surgery, University Medical Center Groningen, Groningen, the Netherlands; 2 Department of Surgery, Maastricht University Medical Center, Maastricht, the Netherlands; 3 Department of General, Visceral and Transplant Surgery, Medizinische Hochschule Hannover, Hannover, Germany; 4 Department of Pediatric Surgery, Academic Medical Center, Amsterdam, the Netherlands; University of Toledo, UNITED STATES

## Abstract

**Background:**

Liver grafts from donation after circulatory death (DCD) donors are increasingly accepted as an extension of the organ pool for transplantation. There is little data on the outcome of liver transplantation with DCD grafts from a pediatric donor. The objective of this study was to assess the outcome of liver transplantation with pediatric DCD grafts and to compare this with the outcome after transplantation of livers from pediatric donation after brain death (DBD) donors.

**Method:**

All transplantations performed with a liver from a pediatric donor (≤16 years) in the Netherlands between 2002 and 2015 were included. Patient survival, graft survival, and complication rates were compared between DCD and DBD liver transplantation.

**Results:**

In total, 74 liver transplantations with pediatric grafts were performed; twenty (27%) DCD and 54 (73%) DBD. The median donor warm ischemia time (DWIT) was 24 min (range 15–43 min). Patient survival rate at 10 years was 78% for recipients of DCD grafts and 89% for DBD grafts (p = 0.32). Graft survival rate at 10 years was 65% in recipients of DCD versus 76% in DBD grafts (p = 0.20). If donor livers in this study would have been rejected for transplantation when the DWIT ≥30 min (n = 4), the 10-year graft survival rate would have been 81% after DCD transplantation. The rate of non-anastomotic biliary strictures was 5% in DCD and 4% in DBD grafts (p = 1.00). Other complication rates were also similar between both groups.

**Conclusions:**

Transplantation of livers from pediatric DCD donors results in good long-term outcome especially when the DWIT is kept ≤30 min. Patient and graft survival rates are not significantly different between recipients of a pediatric DCD or DBD liver. Moreover, the incidence of non-anastomotic biliary strictures after transplantation of pediatric DCD livers is remarkably low.

## Introduction

There is a growing discrepancy between the extensive number of patients waiting for liver transplantation and the availability of organs [1]. Therefore, alternative organ sources have been explored in an effort to increase organ availability. During the last decade, there has been a growing interest in liver donation after circulatory death (DCD), also known as non-heart-beating donation. Most studies report that patient survival after DCD liver transplantation is equivalent to that of DBD liver transplantation. However, graft survival after DCD liver transplantation is lower and rate of primary non-function (PNF), vascular thrombosis, and non-anastomotic biliary strictures is higher than after DBD liver transplantation [2–5]. Despite the less favorable outcome of livers from adult DCD compared to those from adult DBD, the former is accepted as an important source of allografts. The implementation of DCD programs in adults has substantially increased the total number of available livers and thereby reduced waiting list mortality [1,2,6].

Transplantation of DCD donor livers was introduced in the Netherlands in 2001 [7]. The first DCD liver transplantation with a pediatric graft was subsequently performed in 2002. This donor type has the potential to contribute to the number of pediatric organ donors, since withdrawal of life-sustaining therapy accounts for 30–65% of deaths in pediatric intensive care units [8]. A pediatric DCD program may be able to increase the number of donated pediatric livers with 13% to 80% [9]. However, data on the outcome of liver transplantation with DCD grafts from a pediatric donor is limited. Some studies have included a small number of pediatric grafts in general analyses of outcome of DCD liver transplantation, but the outcome of pediatric donor liver grafts has not been reported separately in these studies [2,4,10–13]. There are only thee single center reports of relatively small series of liver transplantation using pediatric DCD grafts [14–17]. These three series include a total number of ten cases.

The aim of this study was to analyze the outcome after transplantation of pediatric (age ≤16 years) DCD liver grafts since the introduction of a national protocol for the procurement of DCD livers in the Netherlands. For this purpose, the outcome after transplantation of pediatric DCD livers was compared with that of pediatric DBD livers in the same time period.

## Materials and methods

### Study design

A retrospective cohort study was performed including all liver transplantations with grafts recovered from pediatric DCD donors aged 16 years or younger in the Netherlands between January 2002 and December 2015. The results of liver transplantation with pediatric DCD grafts were compared with those of pediatric DBD grafts performed in the same time period. The examined parameters included patient survival rate, graft survival rate, and rate of complications including PNF. Follow-up was until August 2016. High urgency and split liver transplantations were excluded since they were only performed with DBD grafts.

### Donor selection

The DCD liver grafts were all procured from controlled donors (Maastricht category III). Liver procurement was cancelled when a) the period between withdrawal of life support and circulatory arrest exceeded one hour, b) the period of hypoperfusion (mean arterial pressure <50 mmHg and saturation <80%) in the donor exceeded 15 minutes, or c) the asystole time (the period between circulatory arrest and start of cold aortic perfusion) exceeded 30 minutes [7,18,19].

### Organ procurement method

The technique of DCD and DBD organ procurement was performed according to national protocol and was described in detail elsewhere [7,18,19]. In summary, in DCD donors with terminal illness or injuries the futile life-sustaining therapy was withdrawn. Preservation measures were started after an obligatory no-touch period of 5 minutes without invasive interventions after circulatory death had been established by an independent physician. Preservation was performed by open aortic cannulation after rapid laparotomy by standby surgical staff. Preservation fluids used to cool and flush the organs were histidine-tryptophan-ketoglutarate (Dr. Franz Köhler Chemie, Bensheim, Germany) or University of Wisconsin solution (Bristol-Myers Squibb B.V., Woerden, The Netherlands), both at 4°C and containing 400 units/kg heparin.

In DBD, brain death was determined according to a standard procedure [18]. An intact circulation during start of organ procurement surgery allowed for preparation time. Once organs were prepared for procurement, donors were systemically heparinized and organ preservation was performed by aortic cannulation, cooling, and flushing with University of Wisconsin solution at 4°C.

In both donor types, after initiating perfusion, the abdominal and thoracic cavity was filled with ice-cold 0.9% sodium chloride solution and slushed ice for topical cooling. Once procured, the livers were packed and stored on melting ice.

### Allocation and transplantation

The Eurotransplant organization allocated the liver grafts to adult and pediatric recipients, according to their position on the waiting list. Centers were allowed to refuse a liver graft, resulting in an allocation to the next recipient on the waiting list. DCD grafts were not transplanted in Germany due to legislation stipulating that organs could only be recovered from DBD donors and DCD transplantation was prohibited. Standard piggy-back orthotopic liver transplantation was performed if possible. Immunosuppressive regimen evolved over the study period and mainly consisted of induction with basiliximab and maintenance immunosuppression with a calcineurin inhibitor (tacrolimus or cyclosporine) and a rapid taper of steroids, either with or without mycophenolate mofetil.

### Study variables

Detailed information regarding the Dutch donors and recipients was obtained from the Dutch Organ Transplant Registry, to which data was prospectively submitted by all organ procurement and transplant centers. The Dutch Transplantation Foundation maintained the registry. The information regarding foreign recipients was obtained from transplant centers within the Eurotransplant region.

Donor characteristics that were collected included age, sex, and cause of death. Graft and preservation information included donor warm ischemia time (DWIT), asystole time, cold ischemia time (CIT), and anastomosis time. DWIT was defined as period between withdrawal of life support and in situ aortic cold perfusion; asystole time was defined as time between circulatory arrest and in situ aortic cold perfusion; CIT was defined as the time between in situ aortic cold perfusion and removal of the liver from the ice-cold preservation fluid for implantation into the recipient. Anastomosis time was defined as the time between removing the liver graft from the cold preservation fluid to revascularization of the liver.

Recipient characteristics that were collected included age, sex, indication for liver transplantation, time spent waiting for a liver transplantation, earlier transplantation, cause of graft loss, cause of recipient death, and complications. MELD (model for end-stage liver disease) score was calculated as laboratory based MELD score with additional points for standard exceptions according to Eurotransplant criteria.

### Outcome parameters

Recipient survival was defined as the time from transplantation to recipient death. Graft survival was defined as the time from transplantation to retransplantation or recipient death. Complications included PNF, infection, hepatic artery thrombosis, portal vein thrombosis, and rejection. PNF was defined as liver failure requiring retransplantation or leading to death within seven days after transplantation without any identifiable cause and other causes of failure such as surgical problems, hepatic artery thrombosis, portal vein thrombosis and acute rejection [20].

### Ethics

Collection, storage and use of patient data were performed in agreement with the ‘Code of Conduct for health research’, put forward by the federation of Dutch medical scientific societies (http://www.federa.org) and conducted in accordance with the Declaration of Helsinki. This study was approved by the Dutch Transplantation Foundation which is responsible for maintaining the Dutch Organ Transplant Registry. This type of research is compliant with Dutch legislation and this study was retrospectively approved by the Medical Ethical Review Board of the University Medical Center Groningen. The reference number of the approval statement is M16.204932. None of the transplant donors were from a vulnerable population and all donors or next of kin provided informed consent that was freely given.

### Statistical analysis

Statistical analysis was performed using SPSS 22.0 for Windows (SPSS Inc., Chicago, IL). Data was presented as median with interquartile range in parenthesis or as number with percentages. Continuous data was compared with Mann-Whitney U test and proportions with Fisher’s exact or chi square test, when appropriate. Graft and recipient survival analyses were determined with the Kaplan-Meier method and significance of survival differences was determined with the log rank test. Rates of complications were compared between groups with univariate logistic regression analysis. Tests were all 2-sided and p-values less than 0.05 were considered statistically significant.

## Results

Between 2002 and 2015, a total number of 74 liver transplantations with pediatric grafts were performed with 20 (27%) DCD and 54 (73%) DBD grafts. The median follow-up of functioning grafts was 85 months (43–125 months), 36 months (24–113 months) for the DCD group and 93 months (61–126 months) for the DBD group. The minimum follow-up was 8 months.

### Donor and recipient characteristics

Donor, preservation, graft and recipient characteristics are summarized in Table 1. As expected the donor risk index was higher in the DCD grafts than in the DBD grafts [21]. The median DWIT and asystole time of the DCD grafts was 24 minutes (20–30 minutes) and 16 minutes (11–19 minutes) respectively. Interestingly, the age of the recipients of DCD livers was higher than that of recipients of DBD livers (median of 53 years versus 15 years, p = 0.01). Although the total cold ischemia time was lower for DCD grafts compared to the DBD grafts, the total ischemic preservation time was equivalent. All other variables were comparable between the two groups.

**Table 1:** Baseline characteristics.

	DCD donors (n = 20)	DBD donors (n = 54)	p-value
***Donor characteristics***			
Age (years)^y^	14 (3–16)	13 (1–16)	0.10
Age ≤12 years	6 (30%)	27 (50%)	0.19
Sex (male)	13 (65%)	30 (56%)	0.60
Donor weight (kg)^y^	52 (16–95)	45 (10–90)	0.11
Severe head trauma	8 (40%)	23 (43%)	1.00
Latest GGT (U/L)	21 (13–41)	17 (12–30)	0.21
Latest ALT (U/L)	32 (20–81)	45 (24–78)	0.75
Donor risk index	1.80 (1.70–2.07)	1.48 (1.16–1.90)	0.01
***Preservation characteristics***			
Donor warm ischemia time (min)	24 (20–30)^z^	NA	NA
Asystole time (min)	16 (11–19)	NA	NA
Cold ischemia time (min)	458 (388–533)	521 (451–598)	0.04
Anastomosis time (min)	35 (26–44)	38 (30–49)	0.24
Total preservation time (min)^§^	480 (419–553)	521 (451–598)	0.17
***Recipient characteristics***			
Age (years)^y^	53 (0–62)	15 (6–67)	0.01
Age ≤16 years	3 (15%)	30 (56%)	0.02
Sex (male)	11 (55%)	25 (46%)	0.51
MELD score	24 (20–26)^k^	25 (20–31)^¶^	0.35
Earlier transplantation	2 (10%)	9 (17%)	0.47
Duration on waiting list (days)	126 (44–371)	241 (87–399)	0.22

Numbers represent median (interquartile range) or number (percentages). ALT, alanine aminotransferase; DCD, donation after circulatory death; DBD, donation after brain death; MELD, model for end-stage liver disease; NA, not applicable.

^y^ Number represent median (range).

^z^ Excluding one patients with missing values.

^§^ Total preservation time is defined as period between withdrawal of life support and graft reperfusion in the recipient.

^k^ Excluding five patients with missing values.

^¶^ Excluding seventeen patients with missing values.

### Patient survival

**Fig 1:**
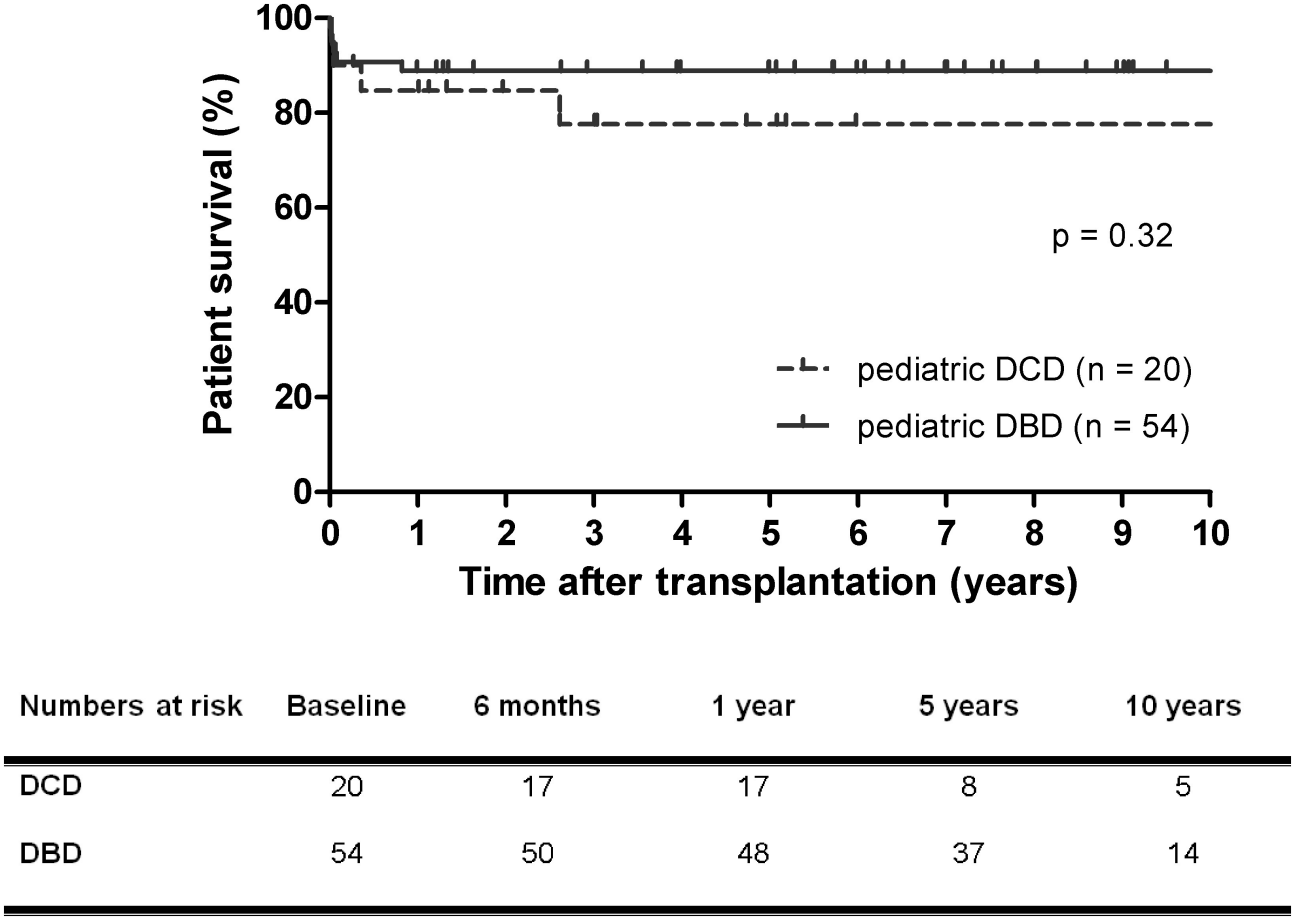
Kaplan-Meier patient survival curves after pediatric DCD and DBD liver transplantation. Patient survival rate of pediatric DCD and DBD liver transplantation was equivalent. DCD, donation after circulatory death, DBD, donation after brain death.

Patient survival rate was similar for recipients of DCD versus DBD liver grafts (78% for DCD versus 89% for DBD at 1 year and 10 years, p = 0.32) (Fig 1). After the first year the survival curves ran virtually parallel up to ten years after transplantation. Four of twenty (20%) recipients of DCD grafts and six of 54 (11%) recipients of DBD grafts died.

### Graft survival

**Fig 2:**
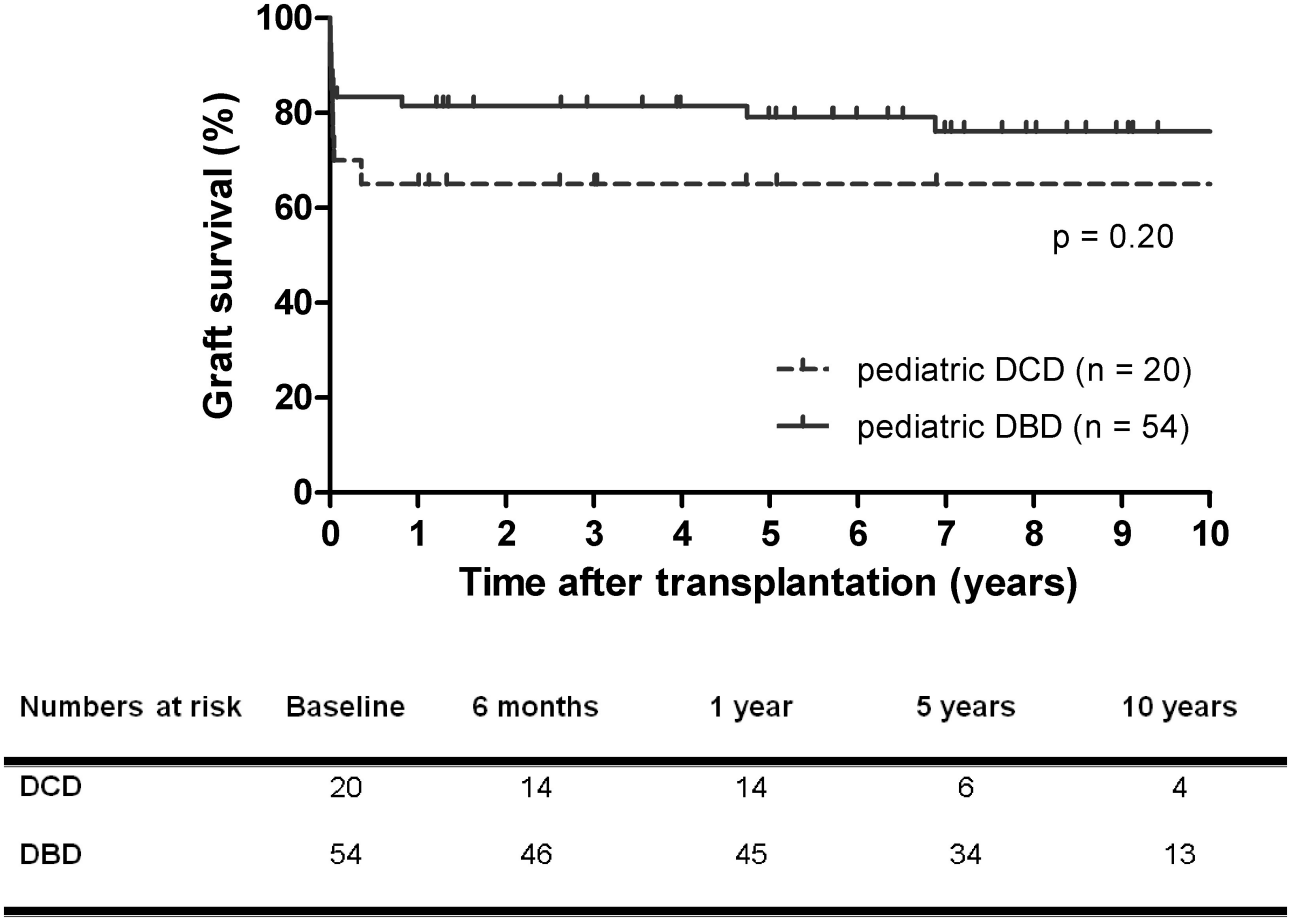
Kaplan-Meier graft survival curves after pediatric DCD and DBD liver transplantation. Graft survival rate of transplantation with pediatric DCD liver grafts was lower than that with pediatric DBD liver grafts, but did not reach statistically significant difference. DCD, donation after circulatory death, DBD, donation after brain death.

Graft survival rate was 65% at 1 year in the DCD group, compared to 82% at 1 year in the DBD group (p = 0.20) (Fig 2). At 10 years, graft survival rate was 65% in recipients of DCD versus 76% in DBD grafts. For grafts functioning after 3 months, 10-year graft survival rate was 93% for DCD grafts versus 91% for DBD grafts (p = 0.71).

Most cases of graft failure (84%) occurred within three months after transplantation. Graft loss occurred in seven of twenty (35%) recipients of DCD grafts and twelve of 54 (22%) recipients of DBD grafts. The etiology of graft loss of the DCD grafts is summarized in Table 2. The graft loss of pediatric DBD livers was due to hepatic artery thrombosis in four patients, PNF in two patients, chronic rejection in one patient, recurrence of primary sclerosing cholangitis in two patients, and patient death in three cases. The majority of recipients with graft failure underwent retransplantation (fourteen of nineteen recipients, 74%), which unfortunately lead to death in a total of six recipients.

**Table 2:** Outcome after liver transplantation with pediatric DCD grafts.

Age donor (years)	Age recipient (years)	Weight donor (kg)	Weight recipient (kg)	Donor warm ischemia time (min)	Asystole time (min)	Graft failure	Etiology of graft failure	Graft survival (months)	Recipient death
3	6	24	19	20	6	Yes	Hepatic artery thrombosis	0.2	Yes
3	7	16	29	24	16	Yes	Hepatic artery thrombosis	0.6	No
9	13	30	48	31	14	Yes	Portal vein thrombosis	0.3	No
11	55	38	65	22	11	No		60.9	No
12	30	32	70	≥33^y^	33	Yes	Primary non function	0.0	No
12	56	45	69	30	16	No		144.9	No
13	64	60	71	17	10	No		31.3	Yes
13	56	50	90	15	10	No		36.4	No
13	44	51	65	28	19	No		31.3	No
14	55	70	93	17	10	No		82.7	No
14	63	72	72	24	12	No		56.8	No
14	67	50	60	24	20	No		15.9	No
15	31	50	80	26	20	Yes	Bile leak, sepsis, and multiorgan failure	4.3	Yes
15	48	60	102	29	18	No		147.7	No
15	56	53	66	27	17	No		13.5	No
16	57	95	90	43	28	Yes	Primary non function	0.1	Yes
16	50	70	89	35	17	Yes	Hepatic artery thrombosis	0.4	No
16	51	70	65	30	13	No		143.7	No
16	64	60	93	19	13	No		36.1	No
16	44	60	76	20	15	No		12.1	No

^y^ The donor warm ischemia time for this liver was missing. However, per definition, it was more than the asystole time which was 33 minutes.

In the DCD group, the DWIT exceeded 30 minutes in four patients. Graft failure occurred in 100% of patients who received a DCD liver with DWIT ≥30 minutes, whereas it occurred in 19% of patients who received a DCD livers with DWIT <30 minutes (p = 0.007). At present the DWIT is kept below 30 minutes as it was recently reported that an extended DWIT of more than 30 minutes is associated with a significantly increased risk of graft failure [22,23]. If donor livers in this study would have been rejected for transplantation when the DWIT was more than 30 minutes, four cases of DCD transplantation would have been excluded from the series. Consequently, the graft failure rate would have been lower: three of sixteen (19%) instead of seven of twenty (35%) liver transplantations. The graft survival rates would have been 81% at 1 year in DCD grafts versus 82% in DBD grafts (p = 0.84).

### Postoperative outcome

The rate of complications within the first year after transplantation was not different between DCD recipients and DBD recipients (Table 3). PNF occurred in two (10%) DCD grafts and two (4%) DBD graft resulting in an odds ratio of 2.9 (95% confidence interval 0.4–22.0) (p = 0.31). Arterial thrombosis occurred in 3 (15%) of DCD grafts and 4 (7%) of DBD grafts resulting in an odds ratio of 2.2 (95% confidence interval 0.4–10.9) (p = 0.33).

**Table 3:** Complications within one year after transplantation.

Complication type	DCD donors (n = 20)	DBD donors (n = 54)	P-value
Primary non-function	2 (10%)	2 (4%)	0.29
Infection	8 (40%)	18 (33%)	1.00
Cardiopulmonary	2 (10%)	3 (6%)	0.61
Neurological	3 (15%)	2 (4%)	0.12
Rejection	0	7 (13%)	0.18
Venous thrombosis	1 (5%)	2 (4%)	0.61
Arterial thrombosis	3 (15%)	4 (7%)	0.38
Non-anastomotic biliary strictures	1 (5%)	2 (4%)	1.00
Anastomotic biliary strictures	2 (10%)	7 (13%)	1.00

Data are presented as number (percentage). DCD, donation after circulatory death; DBD, donation after brain death.

## Discussion

This multicenter study with the largest series of pediatric DCD liver transplantation reports good long-term outcome with 78% patient survival and 65% graft survival at 10 years after transplantation. Patient survival, graft survival, and complication rates were similar between recipients of a pediatric DCD or DBD liver. Moreover, the observed rate of biliary complications and NAS after transplantation of DCD liver grafts was relatively low and no differences were noted between pediatric DCD and DBD livers.

The patient survival rate of pediatric DCD livers in the current study was in line with that of adult DCD liver grafts (78% versus 80–92% at 1 year respectively) [2–5,12,24–26]. However, the graft survival rate of pediatric DCD livers in the present study was 65% at 1 year and was lower than in the pediatric DBD livers in this study (82%). Also, the graft survival rate of pediatric DCD livers in this study was lower than that reported in adult DCD liver transplantation (65% versus 67–79% at 1 year respectively) [2–5,12,24–26]. The cause of graft failure in the pediatric DCD livers in this study was mainly due to vascular complications and PNF. Remarkably, in four DCD liver grafts the DWIT exceeded 30 minutes and these grafts failed after transplantation. At present, DCD livers with DWIT ≥30 minutes are not accepted for transplantation due to a recently demonstrated strong association between DWIT and graft failure after DCD liver transplantation [23,27]. If the four DCD livers with DWIT ≥30 minutes would have been declined for transplantation, the graft survival rate at 1 year would have been 81% which would have been identical to the graft survival rate in the pediatric DBD grafts in the current study (82%). Furthermore, the graft survival in the pediatric DCD grafts would have compared favorably with previous studies of adult DCD liver transplantations [2–5,12,24–26].

In comparison with the current study, the graft and patient survival rates were higher in the previously reported ten cases of transplantation of pediatric DCD grafts (100% in the UCLA group [n = 7] and in the Birmingham group [n = 3]) [14–17]. The high survival rates in these previously reported single center studies may be due to considerably shorter median DWIT (14 min versus 24 min) and CIT (6 hours versus 8 hours) in the Birmingham group compared to the current study, as well as considerably shorter median CIT (5 hours versus 8 hours) in the UCLA group compared to the current study. Furthermore, taking into account the total number of yearly performed liver transplantations in Los Angeles and Birmingham, the low number of reported cases of transplantation of pediatric DCD livers suggests extremely strict selection of recipients and donors in these single center reports (e.g. local donors and negligible DWIT).

In the present study the incidence of NAS in pediatric DCD grafts was relatively low and similar to the incidence in pediatric DBD grafts (5% versus 4% respectively). Interestingly, the incidence of NAS in pediatric DCD livers was considerably lower than that reported in adult DCD livers [19,28–30]. Although it is widely accepted that NAS is the most relevant and prevalent complication of adult DCD livers, this study indicates that this is not the case for pediatric DCD livers. This finding is in line with a recently reported association between NAS and donor age [31]. In transplantation of adult DCD liver grafts the incidence of NAS increases with increasing donor age. Based on a large clinical study, we have recently proposed that impaired biliary regenerative capacity is an important risk factor in the development of NAS [30,32]. The regenerative capacity is in general better preserved in younger age. Altogether these findings indicate that the regenerative capacity is better preserved in younger donors. Therefore, the increased regenerative capacity in young donors may explain the relatively low incidence of NAS observed in this study after liver transplantation of a pediatric DCD graft.

Although the low number of cases warrants careful interpretation of the results of the current study, this study triples the amount of reported transplantations with pediatric DCD liver grafts. Furthermore, the results of pediatric DCD grafts were compared with pediatric DBD grafts to obtain the best estimate of the effect of warm ischemia on these relatively small size pediatric livers. However, as result of small group size a multivariable analysis was not appropriate and survival analyses could not be corrected for differences in baseline characteristics. One of the differences in baseline characteristics was the recipient age which was higher in the DCD than in the DBD grafts. In the current study pediatric DCD livers were generally not transplanted in younger recipients with a long life expectancy. The DCD grafts were probably considered as suboptimal organs because long-term graft survival of DCD livers was considered to be inferior to DBD livers. However, in DCD liver transplantation with adult livers, survival rate of grafts functioning after 1 year is equivalent to that of functioning DBD livers, which is illustrated by graft survival curves of DCD grafts that run parallel to that of DBD grafts at 1 year after transplantation [2–5,24]. Also in this study, graft survival curves of pediatric DCD and DBD grafts run parallel after the first year after transplantation. Therefore, we do believe that pediatric DCD liver grafts should no longer be regarded as suboptimal grafts and acceptance of these livers for pediatric recipients seems justifiable.

In conclusion, this paper describes the largest series of liver transplantation with pediatric DCD grafts and triples the number of reported cases. The results of this multicenter study demonstrate good long-term patient and graft survival rates after transplantation of pediatric DCD livers, especially when DWIT is limited to 30 minutes. Also, the results of this study indicate that risk of NAS is relatively low in pediatric DCD liver grafts. These are important findings in the current era of organ shortage and high mortality rate on the waiting list.

## Abbreviations

ALT: alanine transaminase
CIT: cold ischemia time
DBD: donation after brain death
DCD: donation after circulatory death
DWIT: donor warm ischemia time
MELD: model for end-stage liver disease
NA: not applicable
NAS: non-anastomotic biliary strictures
PELD: pediatric end-stage liver disease
PNF: primary non-function
